# Impact of the COVID-19 pandemic on out-of-hospital cardiac arrest outcomes in older adults in Japan

**DOI:** 10.1016/j.resplu.2022.100299

**Published:** 2022-09-06

**Authors:** Sanae Hosomi, Ling Zha, Kosuke Kiyohara, Tetsuhisa Kitamura, Sho Komukai, Tomotaka Sobue, Jun Oda

**Affiliations:** a Department of Traumatology and Acute Critical Medicine, Osaka University Graduate School of Medicine, 2-15, Yamada-oka, Suita 565-0871, Japan; b Division of Environmental Medicine and Population Sciences, Department of Social Medicine, Graduate School of Medicine, Osaka University, 2-2 Yamada-oka, Suita 565-0871, Japan; c Department of Food Science, Faculty of Home Economics, Otsuma Women’s University, 12 Sanban-cho, Chiyoda-ku, Tokyo 102-8357, Japan; d Division of Biomedical Statistics, Department of Integrated Medicine, Graduate School of Medicine, Osaka University, 2-2, Yamada-oka, Suita 565-0871, Japan

**Keywords:** Out-of-hospital cardiac arrest, Survival outcomes, Older adults, COVID-19 pandemic

## Abstract

**Aim:**

The coronavirus disease (COVID-19) pandemic has negatively affected access to healthcare and treatment. This study aimed to explore the impact of the COVID-19 pandemic on older adults with out-of-hospital cardiac arrest (OHCA) in Japan, a country with a super-aging society.

**Methods:**

This secondary analysis of the All-Japan Utstein Registry included patients aged 65 years and older with bystander-witnessed OHCA between January 1, 2005, and December 31, 2020. Survival outcomes were compared by time period using multivariable logistic regression analyses. The primary outcome measured was the one-month survival rate with neurologically favorable outcomes.

**Results:**

Before the COVID-19 pandemic, survival outcomes were steadily improving, and 32,024 patients in 2019 and 31,894 in 2020 were eligible for analysis. The proportions of conventional cardiopulmonary resuscitation and shock by public-access automated external defibrillators were lower in 2020 than in 2019 (6.7% versus 5.7%, *p* < 0.001 and 2.5% versus 2.1%, *p* < 0.001, respectively). Compared to 2019, the one-month survival after OHCA and prehospital return of spontaneous circulation decreased significantly in 2020 than in 2019 (7.7% versus 6.6%, adjusted odds ratio [AOR]: 0.88, 95% confidence interval [CI]: 0.83–0.94, and 16.8% versus 14.9%, AOR: 0.87, 95% CI: 0.83–0.91, respectively). The proportion of neurologically favorable outcomes also decreased, but the decrease was not statistically significant (3.4% versus 2.8%, AOR: 0.92, 95% CI: 0.83–1.01).

**Conclusion:**

In this population-focused, bystander-witnessed study regarding OHCA, the analysis of nationwide registry data revealed that the COVID-19 pandemic was associated with reduced survival among older adults with OHCA in Japan.

## Introduction

Out-of-hospital cardiac arrest (OHCA) is a major cause of death worldwide. The outcomes of OHCA have reportedly worsened since the start of the coronavirus disease (COVID-19) pandemic.1., 2., 3. Undoubtedly, early bystander cardiopulmonary resuscitation (CPR) and defibrillation with a public-access automated external defibrillator (AED) provide patients with OHCA with greatly increased chances of survival. However, reports from areas severely affected by the COVID-19 pandemic have shown a decrease in bystander CPR and survival at hospital discharge compared with the pre-pandemic period.1., 2., 3. The extent and timing of the COVID-19 epidemic, behavioral restrictions such as lockdowns, and the implementation of preventive measures have varied worldwide. In Japan, the first COVID-19 case was reported on January 15, 2020, and there were three epidemic waves in 2020. A state of emergency (SOE) was declared from April 7 to May 25, 2020, to control its spread. The behavioral restrictions were not as strict as those implemented during lockdowns in some other countries.

Most OHCA among older adults are associated with a poor prognosis, such as non-shockable rhythms, unwitnessed arrest, and no bystander CPR.^4^ For example, in Japan most adults aged 75 years or older who experience an OHCA have asystole as the initial rhythm. Consequently, the outcome of OHCA resuscitation in older individuals is poor. Considering that older adults (aged ≥ 65 years) have been the most adversely affected by COVID-19 and account for >80% of OHCA,^5^ this study aimed to explore the impact of the COVID-19 pandemic on OHCA in older adults in Japan using data from the All-Japan Utstein Registry, a nationwide prospective, population-based OHCA registry. Therefore, in this study, after confirming the trend of OHCA survival since 2013, we analyzed whether they were affected by the COVID-19 pandemic and further focused on specific periods, including the SOE or non-SOE periods.

## Methods

### Study design and setting

The All-Japan Utstein Registry is a prospective, population-based OHCA registry based on the standardized Utstein style.6., 7. This observational study included older adults (aged ≥ 65 years) with bystander-witnessed OHCA of cardiac or noncardiac origin in whom resuscitation was attempted by citizens or emergency medical service (EMS), and who were transported to medical institutions between January 1, 2005, and December 31, 2020. EMS-witnessed and non-witnessed cases and children were excluded because their characteristics and outcomes differ.^8^ We also excluded cases with missing outcomes or variables required for the multivariable logistic regression. Cardiac arrest was defined as the cessation of cardiac mechanical activity, confirmed by the absence of signs of circulation.9., 10. In this registry, cardiac arrests were classified into those presumed to be of cardiac or noncardiac origin, with the latter resulting from cerebrovascular disease, asphyxia, malignant tumors, external causes, drug overuse, anaphylaxis, accidental hypothermia, traffic collision, and other causes. These clinical diagnoses were made by the physician in charge, who worked in collaboration with the EMS personnel.

### Emergency medical service organization in Japan

Details of the EMS system in Japan have been described previously.9., 10., 11. EMS providers are not permitted to terminate resuscitation in the field. The use of AEDs by citizens has been legally permitted since July 2004. All EMS providers perform CPR according to the Japanese CPR guidelines.^12^ In Japan, approximately 2 million citizens participate in community CPR programs, which include training in chest compression, mouth-to-mouth ventilation, and AED use.10., 13. During the COVID-19 pandemic, in addition to standard precautions, EMS personnel were required to wear N95 face masks and isolation gowns when attending to patients with OHCA. Since April 24, 2020, the EMS protocol has encouraged paramedics to use supraglottic airway management instead of endotracheal intubation. In addition, when the SOE was declared, fire departments suspended the provision of CPR training to the public.

### Data collection and quality control

Data were collected prospectively using a form that included data recommended in the Utstein-style reporting guidelines for cardiac arrest.6., 7. All survivors who experienced an OHCA were followed up for up to 1 month after the event by the EMS personnel in charge. One-month neurological outcomes were determined by the physician responsible for treating the patient using the cerebral performance category (CPC) scale, which is measured as follows: category 1, good cerebral performance; category 2, moderate cerebral disability; category 3, severe cerebral disability; category 4, coma or vegetative state; and category 5, death.6., 7.

### Outcome measures

The primary outcome measure was the one-month survival rate with neurologically favorable outcomes (CPC category 1 or 2).6., 7. The secondary outcome measures were prehospital return of spontaneous circulation (ROSC) and the one-month survival rate.

### Statistical analysis

Categorical variables are summarized as counts with proportions, and the χ^2^ test was used to evaluate differences between groups. Continuous variables are summarized as medians with interquartile ranges, and the Wilcoxon–Mann–Whitney U test was used to evaluate differences between groups.

The annual trends in each outcome from 2013 to 2020 were assessed using linear trend tests. Factors associated with one-month survival, prehospital ROSC, and neurologically favorable outcomes, were assessed using multivariable logistic regression to calculate the adjusted odds ratios (ORs) and 95% confidence intervals (CIs). Factors that were considered to be associated with clinical outcomes were included as potential founders in the multivariable logistic regression analyses.8., 9., 10., 11. These variables included age (65–74/75–84/≥85 years), sex, witness status (witnessed by a family member/non-family member), presumed cardiac cause (yes/no), first documented rhythm (ventricular fibrillation/pulseless ventricular tachycardia/pulseless electrical activity/asystole), use of an AED (yes/no), bystander CPR status (chest compression only/chest compression with breathing rescue/no CPR), advanced airway management (endotracheal intubation/supraglottic airway/none), epinephrine (yes/no), EMS response time (call time to patient contact), daytime (9:00 a.m.–4:59 p.m.) (yes/no), weekend/holiday (yes/no), and dispatcher instruction (yes/no).

All statistical analyses were conducted using Stata, version 16 (StataCorp LP, College Station, TX, USA). All tests were two-tailed, and *p*-values of <0.05 were considered statistically significant. This manuscript complies with the STROBE statement for the reporting of cohort and cross-sectional studies.^14^

## Results

**Fig. 1:**
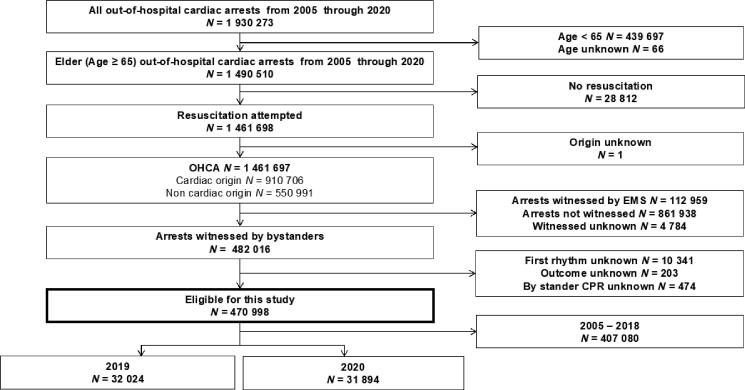
Flow chart of patient selection, Patients aged 65 years or older with bystander-witnessed out-of-hospital cardiac arrests in Japan during 2005–2020 were included in this study. COVID-19, coronavirus disease; EMS, emergency medical service; OHCA, out-of-hospital cardiac arrest.

During the study period, 1,490,510 cases of bystander-witnessed OHCA in older adults were documented, of which 470,998 cases (407,080 in 2005–2018; 32,024 in 2019; and 31,894 in 2020) were eligible for inclusion in the analysis after the exclusion of the following cases: first rhythm unknown (*N* = 10,341), outcome unknown (*N* = 203), bystander CPR unknown (*N* = 474) (Fig. 1).

**Fig. 2:**
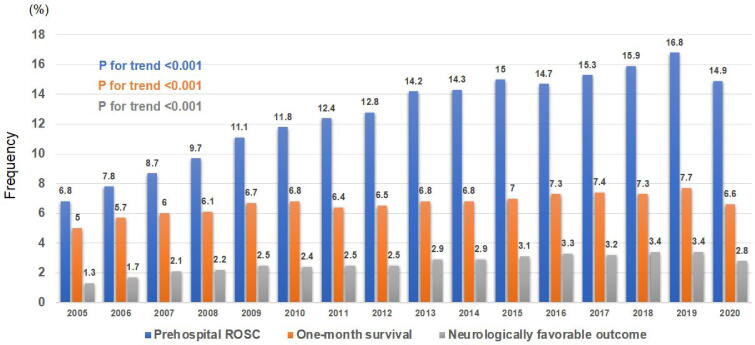
Trend of survival outcomes in this study, the trend bars are based on an analysis of 1,490,510 patients aged 65 years or older with bystander-witnessed out-of-hospital cardiac arrest during 2005–2020. *P*-values for trend were included in the analysis. ROSC, return of spontaneous circulation.

**Fig. 3:**
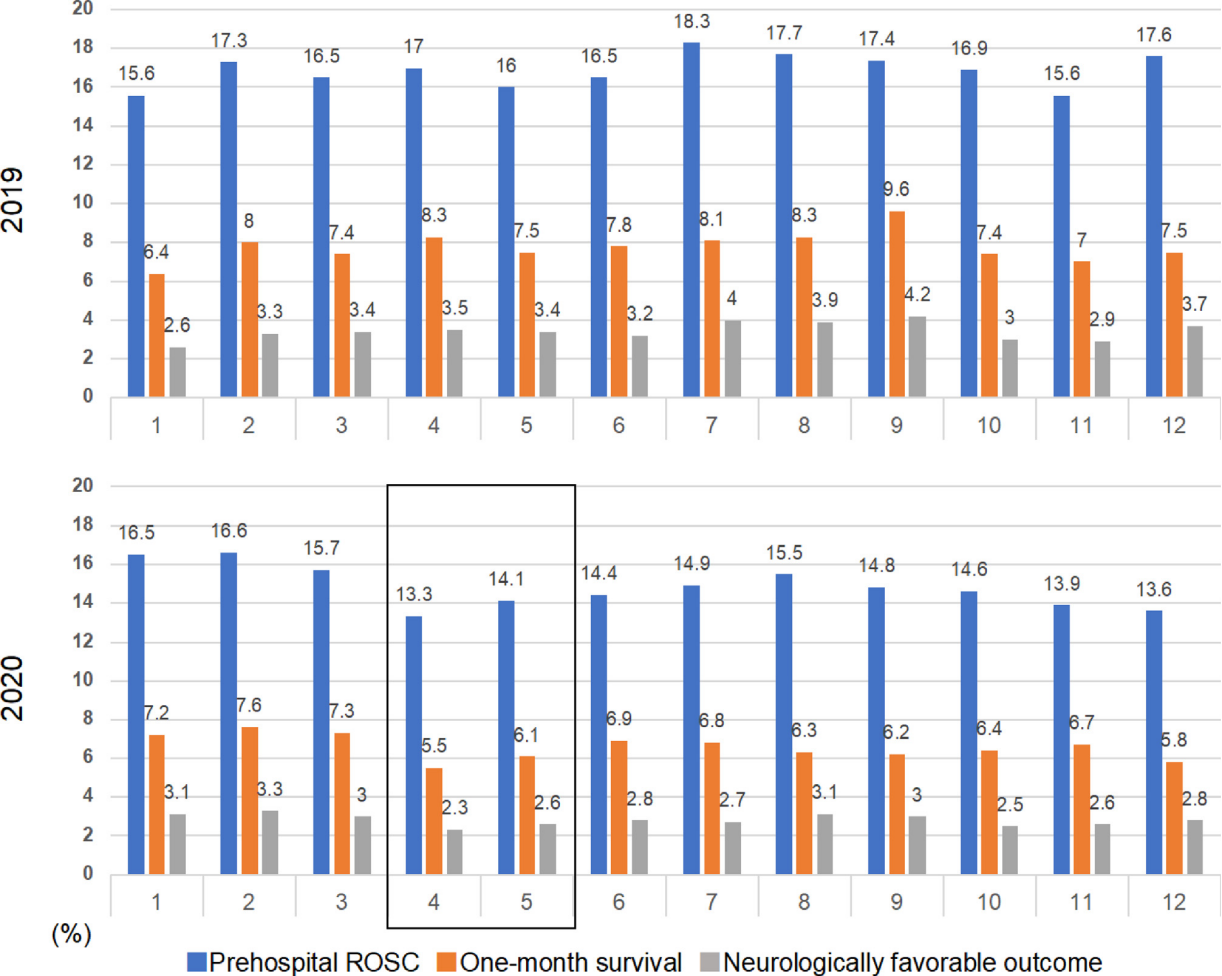
Outcomes of patients with bystander-witnessed out-of-hospital cardiac arrest by month in 2019 and 2020 with the state of emergency period highlighted using a box, ROSC, return of spontaneous circulation.

Before the COVID-19 pandemic, all survival outcomes were steadily improving. The prehospital ROSC more than doubled from 6.8% in 2005 to 14.9% in 2020 (*P_trend_* < 0.001, Fig. 2). Additionally, the one-month survival rate improved from 5.0% in 2005 to 6.6% in 2020 (*P_trend_* < 0.001, Fig. 2). The incidence rate per 100,000 persons was 89.3 in 2019 and 88.2 in 2020. The characteristics of older adults with OHCA in 2019 and 2020 are shown in Table 1. The proportions of OHCA of cardiac origin and witnessed by a family member were significantly higher in 2020 than in 2019 (60.7% in 2019 versus 62.2% in 2020, *p* < 0.001 for family member witness: 61.8% in 2019 versus 63.1% in 2020 for cardiac origin, respectively). Conversely, the proportions of conventional CPR and shock by public-access AEDs were lower in 2020 than in 2019. Emergency response time was longer, and the proportion of dispatcher instruction was higher in 2020 than 2019. Moreover, the change during the SOE (April and May) was observed by the proportion of shocks administered by the public-access AEDs (Supplemental Tables 1 and 2). The survival rate was low in 2020, especially during the SOE (April and May) (Fig. 3).

**Table 1:** Characteristics of patients aged 65 years or older with bystander-witnessed out-of-hospital cardiac arrests in Japan in 2019 and 2020.

		Total	2019	2020	*p*-value
		*N* = 63,918	*N* = 32,024	*N* = 31,894	
Sex	Male (%)	36,311 (56.8%)	18,116 (56.6%)	18,195 (57.0%)	0.22
Age, years, median (IQR)		83 (75–89)	83 (75–89)	83 (76–89)	0.31
Age group (years)	65–74	14,372 (22.5%)	7,275 (22.7%)	7,097 (22.3%)	0.17
	75–84	22,771 (35.6%)	11,305 (35.3%)	11,466 (36.0%)	
	>85	26,775 (41.9%)	13,444 (42.0%)	13,331 (41.8%)	
Type of bystander-witnessed status, n (%)	Family member	39,256 (61.4%)	19,433 (60.7%)	19,823 (62.2%)	<0.001
Origin of arrest, n (%)	Cardiac origin	39,937 (62.5%)	19,806 (61.8%)	20,131 (63.1%)	<0.001
Initial rhythm, n (%)	VF/pVT	6,858 (10.7%)	3,514 (11.0%)	3,344 (10.5%)	0.14
	PEA	25,059 (39.2%)	12,517 (39.1%)	12,542 (39.3%)	
	Asystole	32,001 (50.1%)	15,993 (49.9%)	16,008 (50.2%)	
Type of bystander-initiated CPR, n (%)	Chest compression–only CPR	31,822 (49.8%)	15,704 (49.0%)	16,118 (50.5%)	<0.001
	Conventional CPR with chest compressions and rescue breathing	3,948 (6.2%)	2,143 (6.7%)	1,805 (5.7%)	
	None	28,148 (44.0%)	14,177 (44.3%)	13,971 (43.8%)	
Shocks by public-access AEDs, n (%)		1,479 (2.3%)	811 (2.5%)	668 (2.1%)	<0.001
Advanced airway management, n (%)	Endotracheal intubation	6,205 (9.7%)	3,111 (9.7%)	3,094 (9.7%)	<0.001
	Supraglottic airway	23,681 (37.0%)	11,421 (35.7%)	12,260 (38.4%)	
	None	34,032 (53.2%)	17,492 (54.6%)	16,540 (51.9%)	
Epinephrine, n (%)		22,919 (35.9%)	11,297 (35.3%)	11,622 (36.4%)	0.002
Response time, min, median (IQR)		9 (7–11)	9 (7–11)	9 (7–11)	<0.001
Hospital arrival time, min, median (IQR)		33 (27–40)	32 (27–40)	33 (27–41)	<0.001
Holiday, n (%)		21,697 (33.9%)	10,971 (34.3%)	10,726 (33.6%)	0.093
Daytime, n (%)		26,061 (40.8%)	13,096 (40.9%)	12,965 (40.7%)	0.53
Dispatcher instruction, n (%)		37,520 (58.7%)	18,394 (57.4%)	19,126 (60.0%)	<0.001

Abbreviations: AED, automated external defibrillator; CPR, cardiopulmonary resuscitation; IQR, Interquartile range; PEA, pulseless electrical activity; pVT, pulseless ventricular tachycardia; VF, ventricular fibrillation.

Compared to 2019, the one-month survival after an OHCA and prehospital ROSC decreased significantly in 2020 (7.7% versus 6.6%, adjusted OR (AOR): 0.88, 95% CI: 0.83–0.94 and 16.8% versus 14.9%, AOR: 0.87, 95% CI: 0.83–0.91, respectively). Furthermore, compared to 2019, the proportion of patients with neurologically favorable outcomes decreased, albeit not significantly in 2020 (3.4% versus 2.8%, AOR: 0.92, 95% CI: 0.83–1.01; Table 2). In 2020, neurologically favorable outcomes were similar during the non-SOE and SOE periods (2.9% versus 2.5%, AOR: 0.94, 95% CI: 0.74–1.18).

**Table 2:** Survival outcomes of patients aged 65 years or older with bystander-witnessed out-of-hospital cardiac arrests in Japan in 2019 and 2020.

	2019 *N* = 32,024	2020 *N* = 31,894	p-value (2019 vs 2020)	2020 not during the SOE *N* = 27,913	2020 during the SOE *N* = 3,981	*p*-value (not during vs during the SOE)
Neurologically favorable outcome, n (%)	1,078 (3.4%)	906 (2.8%)		807 (2.9%)	99 (2.5%)	
Crude OR (95% CI)	1 (reference)	0.84 (0.77–0.92)	<0.001	1 (reference)	0.86 (0.69–1.06)	0.151
Adjusted OR (95% CI)	1 (reference)	0.92 (0.83–1.01)	0.090	1 (reference)	0.94 (0.74–1.18)	0.571
Prehospital ROSC, n (%)	5,376 (16.8%)	4,743 (14.9%)		4,201 (15.1%)	542 (13.6%)	
Crude OR (95% CI)	1 (reference)	0.87 (0.83–0.90)	<0.001	1 (reference)	0.89 (0.81–0.98)	0.017
Adjusted OR (95% CI)	1 (reference)	0.87 (0.83–0.91)	<0.001	1 (reference)	0.92 (0.83–1.02)	0.104
One-month survival, n (%)	2,457 (7.7%)	2,096 (6.6%)		1,867 (6.7%)	229 (5.8%)	
Crude OR (95% CI)	1 (reference)	0.85 (0.80–0.90)	<0.001	1 (reference)	0.85 (0.74–0.98)	0.026
Adjusted OR (95% CI)	1 (reference)	0.88 (0.83–0.94)	<0.001	1 (reference)	0.89 (0.76–1.03)	0.122

OR, odds ratio; ROSC, return of spontaneous circulation; SOE, state of emergency. The variables included age, sex, witness status, presumed cardiac cause, first documented rhythm, use of an AED, bystander CPR status, advanced airway management, epinephrine, EMS response time, daytime, weekend/holiday, region of accident, and dispatcher instruction. We calculated the area under the receiver operator characteristic curve to determine discrimination for the primary outcome (area under receiver operator characteristic curve = 0.8943).

## Discussion

The nationwide OHCA registry data showed that the COVID-19 pandemic was associated with a reduction in survival outcomes among older adults with bystander-witnessed OHCA in Japan, a country with a rapidly aging population. To the best of our knowledge, this is the first study to report the impact of COVID-19 on the survival outcomes among older adult population for bystander-witnessed OHCA using nationwide registry data in Japan. Interestingly, a study from Osaka, Japan, including adults found that, although both bystander CPR and AED usage was lower during the COVID period, the one-month survival rate with favorable neurological outcomes did not change.^15^ This might be because of different patient characteristics, a younger age group, or regional differences (Osaka had a relatively higher prevalence of COVID-19 than other regions in Japan). Similar findings have been reported from several other countries,1., 2., 3. although the other studies did not focus on older adults.1., 2., 3. Our findings among older adults suggest that the impact of COVID-19 on OHCA is similar to the effect produced in the overall population as described by various studies.1., 2., 3., 16. During the COVID-19 pandemic, an increase in the proportion of non-shockable rhythm, higher occurrence of cardiac arrest at home, lower AED use, more intubation, and delays in emergency response time were also reported.1., 2., 3. Despite the adjustment for variables related to prehospital factors, there was a decrease in the survival outcomes during the COVID-19 pandemic. Therefore, unmeasured factors or the direct impact of COVID-19 may have adversely affected OHCA outcomes. Possible reasons include early termination of resuscitation due to the risk to the treating team, changes in the quality of CPR, and lack of advanced treatment after hospital arrival.^17^ At the beginning of the lockdown due to the COVID-19 pandemic, there were periods of confusion in emergency medical system, during which a reduction in the number of EMS calls and poorer OHCA outcomes were reported.^18^ However, in Japan, there was no change in the OHCA outcomes and patient characteristics other than AED use during the SOE. Indirect effects associated with the SOE in Japan (political influence) are less likely to have affected these results than has been reported elsewhere, such as during the lockdown in Paris.^2^

Generally, the outcome of OHCA resuscitation in older individuals is poor. However, our study and a previous study^4^ of older people in Japan showed that the OHCA survival rate was improving prior to the start of the COVID-19 pandemic. Our results of the pre-COVID trends were collated using data from a nationwide retrospective observational study that was conducted in Japan during 2005–2015.^4^ It showed significant improvement in the patients’ neurological outcomes.^4^ As this improvement has stopped since the start of the COVID-19 pandemic, immediate countermeasures are required to improve cardiac care of this high-risk group during the ongoing COVID-19 pandemic. Continuous monitoring is needed in the post-pandemic era.

This study has some limitations. First, the registry did not provide data on patients’ COVID-19 status at the time of the OHCA or data on in-hospital treatment. Second, our results might not be generalizable to other countries, which have different EMS and medical systems, COVID-19 epidemic characteristics, and policies. Third, our study did not include the prevalence of infection in each prefecture.^19^ Further, detailed research is needed in the future in this regard. Finally, as with all retrospective studies, data integrity, validity, and ascertainment bias are potential limitations.

## Conclusion

In this population-focused, bystander-witnessed study regarding OHCA in Japan, both one-month survival rate and prehospital ROSC after OHCA decreased significantly during the COVID-19 pandemic period. In addition, AED use and conventional CPR with chest compressions and rescue breathing decreased in 2020 than in 2019. Further studies are needed to characterize OHCA-related mortality in older adults during the COVID-19 pandemic relative to historical mortality patterns, taking in-hospital factors into account.

## Data Availability

The data that support the findings of this study are available from the All-Japan Utstein Registry; restrictions apply to the availability of these data, which were used under license for the current study and are, therefore, not publicly available. However, the data are available from the authors upon reasonable request and with permission from the All-Japan Utstein Registry.
