# Supplementary material for: Impact of the COVID-19 pandemic on out-of-hospital cardiac arrest outcomes in older adults in Japan
Source: Resusc Plus. 2022 Sep 6;12:100299. doi: 10.1016/j.resplu.2022.100299 (PMC9444504; doi:10.1016/j.resplu.2022.100299)
Supplement: Supplementary data 1 [file mmc1.docx]

Supplemental Table 1: Characteristics of patients with bystander-witnessed out-of-hospital cardiac arrests for each month in 2019

| **Month** | **1** | **2** | **3** | **4** | **5** | **6** | **7** | **8** | **9** | **10** | **11** | **12** | ***p*-value** |
| --- | --- | --- | --- | --- | --- | --- | --- | --- | --- | --- | --- | --- | --- |
|  | **N = 4,134** | **N = 2,891** | **N = 2,817** | **N = 2,572** | **N = 2,327** | **N = 2,085** | **N = 2,157** | **N = 2,234** | **N = 2,200** | **N = 2,509** | **N = 2,889** | **N = 3,209** |  |
| **Sex, male, n (%)** | 2,392 (57.9%) | 1,588 (54.9%) | 1,642 (58.3%) | 1,436 (55.8%) | 1,307 (56.2%) | 1,161 (55.7%) | 1,213 (56.2%) | 1,264 (56.6%) | 1,236 (56.2%) | 1,386 (55.2%) | 1,642 (56.8%) | 1,849 (57.6%) | 0.22 |
| **Age, years, median (IQR)** | 83 (76–89) | 83 (75–89) | 83 (75–88) | 82 (75–88) | 82 (75–88) | 83 (75–89) | 83 (75–88) | 82 (75–89) | 82 (75–89) | 83 (75–89) | 83 (76–89) | 83 (75–89) | 0.46 |
| **Age group (years)** |  |  |  |  |  |  |  |  |  |  |  |  |  |
| 65–74 | 889 (21.5%) | 631 (21.8%) | 654 (23.2%) | 585 (22.7%) | 545 (23.4%) | 491 (23.5%) | 528 (24.5%) | 551 (24.7%) | 480 (21.8%) | 596 (23.8%) | 615 (21.3%) | 710 (22.1%) | 0.074 |
| 75–84 | 1,444 (34.9%) | 1,026 (35.5%) | 981 (34.8%) | 941 (36.6%) | 828 (35.6%) | 708 (34.0%) | 737 (34.2%) | 767 (34.3%) | 816 (37.1%) | 850 (33.9%) | 1,064 (36.8%) | 1,143 (35.6%) |  |
| ≥85 | 1,801 (43.6%) | 1,234 (42.7%) | 1,182 (42.0%) | 1,046 (40.7%) | 954 (41.0%) | 886 (42.5%) | 892 (41.4%) | 916 (41.0%) | 904 (41.1%) | 1,063 (42.4%) | 1,210 (41.9%) | 1,356 (42.3%) |  |
| **Type of bystander-witnessed status, family member, n (%)** | 2,709 (65.5%) | 1,796 (62.1%) | 1,684 (59.8%) | 1,498 (58.2%) | 1,416 (60.9%) | 1,219 (58.5%) | 1,264 (58.6%) | 1,371 (61.4%) | 1,295 (58.9%) | 1,480 (59.0%) | 1,750 (60.6%) | 1,951 (60.8%) | <0.001 |
| **Origin of arrest, cardiac origin, n (%)** | 2,514 (60.8%) | 1,802 (62.3%) | 1,765 (62.7%) | 1,592 (61.9%) | 1,502 (64.5%) | 1,263 (60.6%) | 1,327 (61.5%) | 1,364 (61.1%) | 1,367 (62.1%) | 1,562 (62.3%) | 1,766 (61.1%) | 1,982 (61.8%) | 0.28 |
| **Initial rhythm, n (%)** |  |  |  |  |  |  |  |  |  |  |  |  |  |
| VF/pVT | 362 (8.8%) | 291 (10.1%) | 300 (10.6%) | 296 (11.5%) | 277 (11.9%) | 260 (12.5%) | 268 (12.4%) | 248 (11.1%) | 263 (12.0%) | 289 (11.5%) | 311 (10.8%) | 349 (10.9%) | <0.001 |
| PEA | 1,586 (38.4%) | 1,122 (38.8%) | 1,179 (41.9%) | 997 (38.8%) | 918 (39.4%) | 808 (38.8%) | 800 (37.1%) | 837 (37.5%) | 832 (37.8%) | 1,017 (40.5%) | 1,138 (39.4%) | 1,283 (40.0%) |  |
| Asystole | 2,186 (52.9%) | 1,478 (51.1%) | 1,338 (47.5%) | 1,279 (49.7%) | 1,132 (48.6%) | 1,017 (48.8%) | 1,089 (50.5%) | 1,149 (51.4%) | 1,105 (50.2%) | 1,203 (47.9%) | 1,440 (49.8%) | 1,577 (49.1%) |  |
| **Type of bystander-initiated CPR, n (%)** |  |  |  |  |  |  |  |  |  |  |  |  |  |
| Chest compression–only CPR | 2,002 (48.4%) | 1,379 (47.7%) | 1,366 (48.5%) | 1,269 (49.3%) | 1,151 (49.5%) | 1,058 (50.7%) | 1,062 (49.2%) | 1,085 (48.6%) | 1,050 (47.7%) | 1,263 (50.3%) | 1,435 (49.7%) | 1,584 (49.4%) | 0.37 |
| Conventional CPR with chest compressions and rescue breathing | 253 (6.1%) | 197 (6.8%) | 183 (6.5%) | 175 (6.8%) | 152 (6.5%) | 147 (7.1%) | 170 (7.9%) | 159 (7.1%) | 146 (6.6%) | 170 (6.8%) | 171 (5.9%) | 220 (6.9%) |  |
| None | 1,879 (45.5%) | 1,315 (45.5%) | 1,268 (45.0%) | 1,128 (43.9%) | 1,024 (44.0%) | 880 (42.2%) | 925 (42.9%) | 990 (44.3%) | 1,004 (45.6%) | 1,076 (42.9%) | 1,283 (44.4%) | 1,405 (43.8%) |  |
| Shocks by public-access AEDs, n (%) | 71 (1.7%) | 56 (1.9%) | 76 (2.7%) | 79 (3.1%) | 64 (2.8%) | 53 (2.5%) | 69 (3.2%) | 47 (2.1%) | 48 (2.2%) | 71 (2.8%) | 82 (2.8%) | 95 (3.0%) | 0.002 |
| **Advanced airway management, n (%)** |  |  |  |  |  |  |  |  |  |  |  |  |  |
| Endotracheal intubation | 429 (10.4%) | 284 (9.8%) | 283 (10.0%) | 218 (8.5%) | 212 (9.1%) | 208 (10.0%) | 192 (8.9%) | 204 (9.1%) | 214 (9.7%) | 237 (9.4%) | 305 (10.6%) | 325 (10.1%) | 0.29 |
| Supraglottic airway | 1,468 (35.5%) | 1,046 (36.2%) | 1,015 (36.0%) | 948 (36.9%) | 813 (34.9%) | 737 (35.3%) | 739 (34.3%) | 783 (35.0%) | 790 (35.9%) | 862 (34.4%) | 1,042 (36.1%) | 1,178 (36.7%) |  |
| Non | 2,237 (54.1%) | 1,561 (54.0%) | 1,519 (53.9%) | 1,406 (54.7%) | 1,302 (56.0%) | 1,140 (54.7%) | 1,226 (56.8%) | 1,247 (55.8%) | 1,196 (54.4%) | 1,410 (56.2%) | 1,542 (53.4%) | 1,706 (53.2%) |  |
| Epinephrine, n (%) | 1,493 (36.1%) | 1,023 (35.4%) | 1,011 (35.9%) | 913 (35.5%) | 810 (34.8%) | 735 (35.3%) | 734 (34.0%) | 731 (32.7%) | 757 (34.4%) | 898 (35.8%) | 1,055 (36.5%) | 1,137 (35.4%) | 0.30 |
| **Response time, min, median (IQR)** | 9 (7–11) | 9 (7–11) | 9 (7–11) | 9 (7–11) | 9 (7–11) | 9 (7–11) | 9 (7–11) | 9 (7–11) | 9 (7–11) | 9 (7–11) | 9 (7–11) | 9 (7–11) | <0.001 |
| **Hospital arrival time, min, median (IQR)** | 33 (27–41) | 32 (27–41) | 32 (26–39) | 32 (27–40) | 32 (26–40) | 32 (26–39) | 32 (26–39) | 33 (27–40) | 33 (26–40) | 33 (26–40) | 33 (27–40) | 33 (27–40) | 0.003 |
| **Holiday, n (%)** | 1,391 (33.6%) | 1,000 (34.6%) | 1,037 (36.8%) | 864 (33.6%) | 949 (40.8%) | 725 (34.8%) | 665 (30.8%) | 714 (32.0%) | 826 (37.5%) | 839 (33.4%) | 1,003 (34.7%) | 958 (29.9%) | <0.001 |
| **Daytime, n (%)** | 1,670 (40.4%) | 1,199 (41.5%) | 1,129 (40.1%) | 1,039 (40.4%) | 946 (40.7%) | 852 (40.9%) | 849 (39.4%) | 958 (42.9%) | 909 (41.3%) | 1,045 (41.7%) | 1,127 (39.0%) | 1,373 (42.8%) | 0.096 |
| **Dispatcher instruction, n (%)** | 2,389 (57.8%) | 1,656 (57.3%) | 1,608 (57.1%) | 1,494 (58.1%) | 1,349 (58.0%) | 1,205 (57.8%) | 1,278 (59.2%) | 1,284 (57.5%) | 1,229 (55.9%) | 1,408 (56.1%) | 1,662 (57.5%) | 1,832 (57.1%) | 0.69 |

Abbreviations: AED, automated external defibrillator; CPR, cardiopulmonary resuscitation; IQR, Interquartile range; PEA, pulseless electrical activity; pVT, pulseless ventricular tachycardia; VF, ventricular fibrillation

Supplemental Table 2: Characteristics of patients with bystander-witnessed out-of-hospital cardiac arrests for each month in 2020, with the SOE period highlighted

| **Month** | **1** | **2** | **3** | **4** | **5** | **6** | **7** | **8** | **9** | **10** | **11** | **12** | ***p*-value** |
| --- | --- | --- | --- | --- | --- | --- | --- | --- | --- | --- | --- | --- | --- |
|  | N = 3,538 | N = 2,823 | N = 2,739 | N = 2,585 | N = 2,327 | N = 2,109 | N = 2,304 | N = 2,323 | N = 2,157 | N = 2,611 | N = 2,844 | N = 3,534 |  |
| **Sex, male, n (%)** | 2,114 (59.8%) | 1,565 (55.4%) | 1,562 (57.0%) | 1,442 (55.8%) | 1,306 (56.1%) | 1,196 (56.7%) | 1,307 (56.7%) | 1,325 (57.0%) | 1,249 (57.9%) | 1,519 (58.2%) | 1,570 (55.2%) | 2,040 (57.7%) | 0.019 |
| **Age, years, median (IQR)** | 83 (76–88) | 83 (76–89) | 83 (75–89) | 83 (76–89) | 83 (76–89) | 82 (75–88) | 82 (75–89) | 82 (75–88) | 83 (76–89) | 82 (75–89) | 83 (76–89) | 83 (76–89) | 0.31 |
| **Age group (years)** |  |  |  |  |  |  |  |  |  |  |  |  |  |
| 65–74 | 769 (21.7%) | 601 (21.3%) | 615 (22.5%) | 563 (21.8%) | 488 (21.0%) | 519 (24.6%) | 545 (23.7%) | 532 (22.9%) | 466 (21.6%) | 594 (22.7%) | 639 (22.5%) | 766 (21.7%) | 0.11 |
| 75–84 | 1,319 (37.3%) | 1,041 (36.9%) | 971 (35.5%) | 887 (34.3%) | 857 (36.8%) | 736 (34.9%) | 820 (35.6%) | 856 (36.8%) | 788 (36.5%) | 927 (35.5%) | 971 (34.1%) | 1,293 (36.6%) |  |
| ≥85 | 1,450 (41.0%) | 1,181 (41.8%) | 1,153 (42.1%) | 1,135 (43.9%) | 982 (42.2%) | 854 (40.5%) | 939 (40.8%) | 935 (40.2%) | 903 (41.9%) | 1,090 (41.7%) | 1,234 (43.4%) | 1,475 (41.7%) |  |
| Type of bystander-witnessed status, family member, n (%) | 2,329 (65.8%) | 1,728 (61.2%) | 1,672 (61.0%) | 1,594 (61.7%) | 1,502 (64.5%) | 1,295 (61.4%) | 1,382 (60.0%) | 1,408 (60.6%) | 1,330 (61.7%) | 1,606 (61.5%) | 1,742 (61.3%) | 2,235 (63.2%) | <0.001 |
| Origin of arrest, cardiac origin, n (%) | 2,175 (61.5%) | 1,794 (63.5%) | 1,744 (63.7%) | 1,656 (64.1%) | 1,504 (64.6%) | 1,305 (61.9%) | 1,456 (63.2%) | 1,427 (61.4%) | 1,364 (63.2%) | 1,684 (64.5%) | 1,761 (61.9%) | 2,261 (64.0%) | 0.10 |
| **Initial rhythm, n (%)** |  |  |  |  |  |  |  |  |  |  |  |  |  |
| VF/pVT | 363 (10.3%) | 270 (9.6%) | 285 (10.4%) | 236 (9.1%) | 250 (10.7%) | 248 (11.8%) | 255 (11.1%) | 257 (11.1%) | 261 (12.1%) | 302 (11.6%) | 293 (10.3%) | 324 (9.2%) | <0.001 |
| PEA | 1,401 (39.6%) | 1,172 (41.5%) | 1,095 (40.0%) | 1,042 (40.3%) | 869 (37.3%) | 817 (38.7%) | 874 (37.9%) | 868 (37.4%) | 827 (38.3%) | 1,055 (40.4%) | 1,154 (40.6%) | 1,368 (38.7%) |  |
| Asystole | 1,774 (50.1%) | 1,381 (48.9%) | 1,359 (49.6%) | 1,307 (50.6%) | 1,208 (51.9%) | 1,044 (49.5%) | 1,175 (51.0%) | 1,198 (51.6%) | 1,069 (49.6%) | 1,254 (48.0%) | 1,397 (49.1%) | 1,842 (52.1%) |  |
| **Type of bystander-initiated CPR, n (%)** |  |  |  |  |  |  |  |  |  |  |  |  |  |
| Chest compression–only CPR | 1,759 (49.7%) | 1,409 (49.9%) | 1,403 (51.2%) | 1,289 (49.9%) | 1,175 (50.5%) | 1,031 (48.9%) | 1,203 (52.2%) | 1,150 (49.5%) | 1,099 (51.0%) | 1,303 (49.9%) | 1,418 (49.9%) | 1,879 (53.2%) | 0.003 |
| Conventional CPR with chest compressions and rescue breathing | 193 (5.5%) | 147 (5.2%) | 159 (5.8%) | 171 (6.6%) | 133 (5.7%) | 131 (6.2%) | 139 (6.0%) | 138 (5.9%) | 134 (6.2%) | 150 (5.7%) | 172 (6.0%) | 138 (3.9%) |  |
| None | 1,586 (44.8%) | 1,267 (44.9%) | 1,177 (43.0%) | 1,125 (43.5%) | 1,019 (43.8%) | 947 (44.9%) | 962 (41.8%) | 1,035 (44.6%) | 924 (42.8%) | 1,158 (44.4%) | 1,254 (44.1%) | 1,517 (42.9%) |  |
| Shocks by public-access AEDs, n (%) | 68 (1.9%) | 66 (2.3%) | 56 (2.0%) | 42 (1.6%) | 40 (1.7%) | 47 (2.2%) | 52 (2.3%) | 54 (2.3%) | 42 (1.9%) | 57 (2.2%) | 65 (2.3%) | 79 (2.2%) | 0.71 |
| **Advanced airway management, n (%)** |  |  |  |  |  |  |  |  |  |  |  |  |  |
| Endotracheal intubation | 402 (11.4%) | 285 (10.1%) | 254 (9.3%) | 215 (8.3%) | 208 (8.9%) | 205 (9.7%) | 208 (9.0%) | 235 (10.1%) | 200 (9.3%) | 233 (8.9%) | 279 (9.8%) | 370 (10.5%) | <0.001 |
| Supraglottic airway | 1,323 (37.4%) | 1,055 (37.4%) | 982 (35.9%) | 926 (35.8%) | 906 (38.9%) | 814 (38.6%) | 903 (39.2%) | 897 (38.6%) | 851 (39.5%) | 1,028 (39.4%) | 1,127 (39.6%) | 1,448 (41.0%) |  |
| Non | 1,813 (51.2%) | 1,483 (52.5%) | 1,503 (54.9%) | 1,444 (55.9%) | 1,213 (52.1%) | 1,090 (51.7%) | 1,193 (51.8%) | 1,191 (51.3%) | 1,106 (51.3%) | 1,350 (51.7%) | 1,438 (50.6%) | 1,716 (48.6%) |  |
| Epinephrine, n (%) | 1,381 (39.0%) | 1,035 (36.7%) | 983 (35.9%) | 929 (35.9%) | 814 (35.0%) | 757 (35.9%) | 828 (35.9%) | 841 (36.2%) | 733 (34.0%) | 956 (36.6%) | 1,040 (36.6%) | 1,325 (37.5%) | 0.033 |
| **Response time, min, median (IQR)** | 9 (7–11) | 9 (7–11) | 9 (7–11) | 9 (7–11) | 9 (8–11) | 9 (8–11) | 9 (8–11) | 9 (8–11) | 9 (8–11) | 9 (8–11) | 9 (8–11) | 9 (8–11) | <0.001 |
| **Hospital arrival time, min, median (IQR)** | 33 (27–40) | 33 (27–40) | 32 (26–39) | 33 (27–40) | 33 (28–40) | 33 (27–40) | 33 (27–40) | 34 (28–41) | 33 (27–40) | 34 (27–41) | 33 (27–41) | 34 (28–42) | <0.001 |
| **Holiday, n (%)** | 1,249 (35.3%) | 1,115 (39.5%) | 890 (32.5%) | 774 (29.9%) | 999 (42.9%) | 589 (27.9%) | 728 (31.6%) | 857 (36.9%) | 726 (33.7%) | 781 (29.9%) | 1,080 (38.0%) | 938 (26.5%) | <0.001 |
| **Daytime, n (%)** | 1,451 (41.0%) | 1,137 (40.3%) | 1,066 (38.9%) | 1,055 (40.8%) | 920 (39.5%) | 848 (40.2%) | 953 (41.4%) | 938 (40.4%) | 892 (41.4%) | 1,060 (40.6%) | 1,196 (42.1%) | 1,449 (41.0%) | 0.65 |
| **Dispatcher instruction, n (%)** | 2,150 (60.8%) | 1,691 (59.9%) | 1,593 (58.2%) | 1,564 (60.5%) | 1,410 (60.6%) | 1,271 (60.3%) | 1,381 (59.9%) | 1,379 (59.4%) | 1,275 (59.1%) | 1,550 (59.4%) | 1,686 (59.3%) | 2,176 (61.6%) | 0.43 |

Abbreviations: AED, automated external defibrillator; CPR, cardiopulmonary resuscitation; IQR, Interquartile range; PEA, pulseless electrical activity; pVT, pulseless ventricular tachycardia; VF, ventricular fibrillation

Supplemental Table 3: Factors associated with neurologically favorable outcome

|  |  | Neurologically favorable outcome | | | Crude odds ratio | | Adjusted odds ratio | | VIF |
| --- | --- | --- | --- | --- | --- | --- | --- | --- | --- |
|  |  | n | N | % |  | (95% CI) |  | (95% CI) |  |
| Year | 2019 | 1,078 | 32,024 | 3.37% | REF |  | REF |  | 1.00 |
|  | 2020 | 906 | 31,894 | 2.84% | 0.84 | 0.77–0.92 | 0.91 | 0.83–1.01 |  |
| Sex | Female | 483 | 27,607 | 1.75% | REF |  | REF |  |  |
|  | Male | 1,501 | 36,311 | 4.13% | 2.42 | 2.18–2.69 | 1.33 | 1.18–1.50 | 1.08 |
| Age group (years) | 65–74 | 1,038 | 14,372 | 7.22% | REF |  | REF |  |  |
|  | 75–84 | 658 | 22,771 | 2.89% | 0.38 | 0.35–0.42 | 0.58 | 0.52–0.65 | 1.70 |
|  | ≥85 | 288 | 26,775 | 1.08% | 0.14 | 0.12–0.16 | 0.26 | 0.23–0.31 | 1.82 |
| Type of bystander-witnessed status | Non-family member | 1,044 | 24,662 | 4.23% | REF |  | REF |  |  |
|  | Family member | 940 | 39,256 | 2.39% | 0.55 | 0.51–0.61 | 0.89 | 0.80–1.00 | 1.18 |
| Origin of arrest | Non-cardiac origin | 338 | 23,981 | 1.41% | REF |  | REF |  |  |
|  | Cardiac origin | 1,646 | 39,937 | 4.12% | 3.01 | 2.67–3.38 | 1.4 | 1.22–1.60 | 1.07 |
| Initial rhythm | VF/pVT | 1,330 | 6,858 | 19.39% | 67.3 | 55.46–81.65 | 33.79 | 27.49–41.54 | 1.45 |
|  | PEA | 540 | 25,059 | 2.15% | 6.16 | 5.03–7.54 | 5.81 | 4.74–7.12 | 1.11 |
|  | Asystole | 114 | 32,001 | 0.36% | REF |  | REF |  |  |
| Type of bystander-initiated CPR | Chest compression–only CPR | 1,125 | 31,822 | 3.54% | 1.54 | 1.40–1.70 | 1.24 | 1.10–1.41 | 1.40 |
|  | Conventional CPR with rescue breathing | 204 | 3,948 | 5.17% | 2.29 | 1.95–2.69 | 1.20 | 0.98–1.47 | 1.18 |
|  | None | 655 | 28,148 | 2.33% | REF |  | REF |  |  |
| Shocks by public-access AEDs | No | 1,540 | 62,439 | 2.47% | REF |  | REF |  |  |
|  | Yes | 444 | 1,479 | 30.02% | 16.96 | 15.01–19.17 | 1.89 | 1.61–2.23 | 1.30 |
| Advanced airway management | Endotracheal intubation | 65 | 6,205 | 1.05% | 0.22 | 0.17–0.28 | 0.45 | 0.34–0.58 | 1.13 |
|  | Supraglottic airway | 360 | 23,681 | 1.52% | 0.32 | 0.29–0.36 | 0.39 | 0.34–0.44 | 1.17 |
|  | Non | 1,559 | 34,032 | 4.58% | REF |  | REF |  |  |
| Epinephrine | No | 1,717 | 40,999 | 4.19% | REF |  | REF |  |  |
|  | Yes | 267 | 22,919 | 1.16% | 0.27 | 0.24–0.31 | 0.34 | 0.29–0.39 | 1.11 |
| Response time | (one-minute increment) |  |  |  | 0.98 | 0.97–0.98 | 0.91 | 0.89–0.93 | 1.03 |
| Holiday | No | 553 | 17,898 | 3.09% | REF |  | REF |  |  |
|  | Yes | 1,328 | 42,221 | 3.15% | 0.96 | 0.87–1.06 | 0.98 | 0.88–1.09 | 1.00 |
| Daytime | No | 656 | 21,697 | 3.02% | REF |  | REF |  |  |
|  | Yes | 1,101 | 26,061 | 4.22% | 1.85 | 1.69–2.02 | 1.24 | 1.12–1.38 | 1.03 |
| Dispatcher instruction, n (%) | No | 851 | 26,398 | 3.22% | REF |  | REF |  |  |
|  | Yes | 1,133 | 37,520 | 3.02% | 0.93 | 0.85–1.02 | 1.01 | 0.91–1.13 | 1.25 |

Abbreviations: AED, automated external defibrillator; CI, confidence interval; CPR, cardiopulmonary resuscitation; PEA, pulseless electrical activity; pVT, pulseless ventricular tachycardia; SOE, state of emergency; VF, ventricular fibrillation; REF, reference; VIF, variance inflation factor
